# An Address Delivered at the First Annual Meeting of the DeWitt County Medical Society, Held at Clinton, Ill. April 6, 1857

**Published:** 1857-06

**Authors:** Christopher Goodbrake


THE NORTH-WESTERN

MEDICAL AND SURGICAL JOURNAL.

(NEW SERIES.)

VOL. VI. JUNE, 1857. NO. 6.

### ORIGINAL COMMUNICATIONS.

ARTICLE I.—*An Address delivered at the first Annual Meeting of the De Witt County Medical Society, held at Clinton, Ill., April 6th*, 1857. By CHRISTOPHER GOODBRAKE, M.D. Published by request of the Society.

*Gentlemen:*—The Constitution we adopted at our first meeting, makes it obligatory upon the President to deliver an address before retiring from the chair. And although, unfortunately for me at this time, I am not 'one of the speaking kind,' I will not shrink from the discharge of this last duty pertaining to the official station, in which your kind partiality has placed me. But to the fulfilment of this obligation I will now devote a brief space of time, and to what I may have to say on this occasion, I beg leave to solicit your kind attention.

The subject which I have not deemed it inappropriate to dwell upon, in what few remarks I shall attempt to make, is a theme worthy the pen of our best writers, and the efforts of our most accomplished speakers in the profession. It is a subject of which we should never allow ourselves to lose sight for a moment, as long as we wish to be considered members of the medical profession, namely: OUR DUTIES AS PHYSICIANS.

There has been a great deal both said and written on this subject; and in the National Code of Ethics, which we have adopted, the duties of the physician are so clearly and ably set forth, that it might seem an act of arrogancy in me, to talk to you on this topic. But I assure you, gentlemen, that I do not wish to be understood as assuming to myself the capability of adding in the least to the excellent precepts laid down for the rule and guide of our conduct, in the finished document referred to. All I will endeavor to do, is, earnestly to direct our attention to the subject, and bring these things home to ourselves, in order, if possible, that our minds may become so familiarized with them that it may became a *habit* with us, on all occasions, to pay especial regard to our obligations as members of the medical fraternity.

Our duties as physicians are manifold and various in their nature, and I will proceed to consider only a few of them, in the order as they may present themselves to my mind.

From the day on which we first open an office, 'hang out our shingle,' and offer our services to the public as members of that noble profession, whose object is to alleviate or remedy the many maladies and diseases incident to the human family,—to the time when we may either quit the profession from choice, or which is far nobler, like the fearless and faithful warrior die at our post; we should most conscientiously avoid every thing, either in word or deed, which might have a tendency to detract from the importance of the science of medicine in the estimation of men; bring reproach upon the profession; unnecessarily or ignorantly, hazard the life and health of any one entrusted to our care; or bring ourselves into deserved disrepute. But, on the contrary, we should do every thing within our power, to promote the advancement of the science of medicine, elevate the character of our profession, exert our best abilities in behalf of our patients, and walk uprightly in our station as physicians before God and man.

If these remarks are correct, and I presume no one will gainsay them; it will be seen that our duties although numerous and diversified, may be grouped under the following four principal heads, namely: *Our duty to the science we profess; Our duties to each other as professional brethren; Our obligations to the community; and the duties we owe to ourselves.*

The science of medicine is a noble science, and worthy the thoughts and labors of the most gifted intellects, in this our day and generation. She has had for her votaries some of the most exalted minds, through a succession of ages, from a very early period in the world's history down to the present time. Such men as Hypocrates, Galen, Celsus, Haller, Sydenham, Cullen, Hunter, Bichat, Rush, Physick, Chapman, Drake, and a host of other worthies, too numerous to mention—all men of giant intellect, have spent their best strength in the upbuilding and fostering of this their favorite science, and have handed it down to us, not indeed perfect, for nothing on earth is perfect; but they have laid the foundation broad and deep upon the adamantine rock of scientific research, cemented it with experimental wisdom, and have reared the *temple of rational medicine* until it rises before our admiring gaze in the most beautiful proportions. And we who wish to be considered as workmen worthy to be engaged in the progressive labor, which still is, and ever will be requisite for the completion and adornment of this stupendous edifice, must strive in some measure at least, and according to the best abilities wherewith God hath blest us, to imitate the noble examples set before us by these *Master-Builders.*

It is our duty then never to relinquish our studies, we must be found at our task early and late. For, if we allow ourselves to became negligent, and cease to refresh our minds at the fountains of science, we will very soon, instead of a regular advance in medical knowledge, find ourselves moving in a retrograde directions. And instead of producing material worthy to be incorporated in the building already referred to, our mark will be found 'neither oblong nor square,' and must necessarily be cast aside among the rubbish, as 'unfit for the builder's use.'

Let us to work then, with willing and ready minds. Without labor we can accomplish nothing; and here I would remark, that our labor is not all disagreeable and uninviting. But I am sorry to say that upon this very rock, so many, *very* many physicians make shipwreck of their scientific faith. As soon as they 'get through,' as they very complacently term it, they cannot think of applying their minds to the head-aching studies of Anatomy, Physiology, Chemistry, or Pathology. And if they study at all, they content themselves with a superficial gleaning of what refers to practice, although it is as certain as the laws of gravitation, that the physician who neglects to keep himself 'posted up' on these fundamental branches, and who lacks the energy to keep pace with the improvements and discoveries, not only in these, but all the other branches, soon becomes unfit even to practice medicine, surgery, obstetrics, &c.; and far less will he have the capacity, or even the inclination, to do any thing for the furtherance of the science he is too indolent to cultivate.

But what is there more entitled to occupy the mind of man than that study which makes him familiar with the great 'Masterpiece' of Gods' handiwork? What more satisfactory than chemistry, which teaches him the elementary principles and properties of his own body, and the material world which surrounds him on all sides? What more beautiful than physiology, which teaches him the laws of life and death? And what more interesting to the philanthropic physician, than the study of the cause, nature, and effects of disease, which furnishes him with data, upon which to institute a judicious treatment, either medical or surgical?

Then let us be up and doing. We can all do something. We may not all become stars of great magnitude in the medical constellation, but we can all contribute our mite however small. And if we do this, not loitering around as loafing worshippers at the shrine of Æculapius—and faithfully discharge our duties, it will not be said of us, after we have gone to—

'That land of deepest shade,
Unpierced by human thought,'

that we have lived in vain, and disgraced the science we professed.

As physicians we should consider each other as belonging to the same family or brotherhood, we should all have the same object in view, and our interests (in a professional sense) should be the same. We should as one man strive for the advancement of the science of medicine, and the elevation of the character of our profession; and this end we can best attain, by first being prepared to discharge the obligations of physicians ourselves, and then act honorably and uprightly in all our intercourse with each other. Nothing has a greater tendency to depreciate the character and usefulness of our profession, in the estimation of the community at large, than our own short-comings in regard to proper qualifications, and the wranglings and backbitings which, I am sorry to say, are far too common among the physicians of some neighborhoods; sometimes it is the case, that one physician has just cause for hard feelings against another. But so far as I have been able to judge, from my own experience and observation, I have been led to the conclusion, that these 'hard feelings' more frequently arise from *imaginary* wrongs. They are not very seldom the offspring of little minds, devoid of magnanimity and nobleness of soul; but full of sickly peevishness and professional jealousy. The physician who is duly qualified to discharge the duties of his office, and who is determined to discharge that duty in a conscientious manner towards his patients, and with due regard to the feelings of his professional neighbors, need scarcely ever be troubled with any of these professional difficulties, as they are usually termed. He will, as a general thing, gain the confidence of the community in which he resides; receive a just reward for all his studies and labors; he will deserve and receive the good will, aid, and comfort of his medical brethren in his own immediate neighborhood, and the esteem of the profession at large, and as a consequence, he can treat with silent contempt the backbitings and *innuendos* of little minded and jealous hearted doctors, and bid defiance to all quackdom, to curtail his practice or injure his reputation as a medical practitioner.

Nevertheless, I will mention one or two of the sources from which 'hard feelings' may arise, and endeavor to explain how they should be met and disposed of. One of these we shall find to rest with the people, who will occasionally have a physician called in to prescribe for one of the family, and probably, as is frequently the case, in this part of the country, inform him that if the patient 'does not get along well, they will let him know.' And then more than likely the very next day, send for a second physician, without any explanation to either. I have frequently known three or four physicians to be called in succession in this manner, sometimes designedly, occasionally maliciously, and very often through ignorance (of the consequences) on the part of those who make these indiscriminate and indiscreet calls on the profession. And in this very way, I have known misunderstandings and ill-feelings to arise among 'the electors,' which it required months, and sometimes years to correct or allay, and which, by a little seasonable 'talk,' might have been all explained, and good will and harmony maintained. When troubles of this kind come to our knowledge, where we ourselves are a party in the case, it is our imperative duty, not to get angry with our brother physician, and go gadding around through town and country, and inform every one we meet, how very mean such or such on one has treated us; but to call on the physician and kindly enquire of him how it happened and he will, as a matter of course, state the case in its proper light. Or, on the other hand, if we were unwittingly caused to encroach upon his rights, it is our duty to make all due explanations to him; and all of us should discontenance such acts on the part of those who employ us. We should teach them what is right and what is wrong, in regard to these things, and this we can do without any hard thoughts on their part whatever. On the contrary, they will esteem us for candor and plain-dealing between our professional brethren and themselves.

Another fruitful source of trouble among physicians, is a spirit of professional officiousness, as it is called by some,—I would call it *un*professional rather. One of the most despicable characteristics in the physician, is an insidious meddlesomeness in another's affairs. Some physicians make it their business to seem to have an extraordinary sympathy for every one who happens to be sick, and who is so unfortunate as not to have secured their services; and if they do not go directly to the patient's room, they accidentally call in to see his friends, and enquire very humanely all about the case, pump out of them all they may know about the symptoms and treatment,—then probably give an ominous shake of the head, or shrug of the shoulder, and enquire if such or such remedies have not been tried. And upon receiving an answer in the negative, will say ''tis strange, 'tis passing strange;' and finally wind up by telling the friends, that they 'hadn't ought to say any thing,'

but they merely wish to intimate, that they had a patient *precisely similarly* affected, and the case was got along with, without any trouble whatever. Now, all this is dastardly mean, I need not tell you it is unprofessional; but it matters not by whom it is practiced, whether they call themselves 'regular' or 'irregular' we need not go to the trouble of calling down anathemas upon their devoted heads, for verily, they will very soon reap their just reward. They will be held in contempt and detestation by all highminded and honorable physicians; and the people soon decerning their object, will withdraw what little patronage they formerly bestowed upon them. I am sure we have none such in our Society, and for the sake of the honor of the profession, I hope we have none in this part of the country—who profess to be regular practitioners, and who would notwithstanding be guilty of such ungentlemanly and reprehensible conduct.

Gentlemen, in the practice of medicine as in all other callings, honesty is the best policy. If we are properly qualified for the practice of our profession, and act honorably in all our dealings with those who employ us, and with one another, I have no fears that our exertions will not be rewarded with a reasonable share of success, both in a professional and pecuniary sense; and we will be spared the labor to withstand the temptation of endeavoring to supplant one another. Let us then deal honorably and uprightly with each other; ever remembering, that every word spoken against a worthy member of our profession, is just so much calumny against the profession at large. If a man practices medicine among us, let us endeavor to treat him as he deserves. If he be a gentleman, worthy to be taken by the hand as a physician, never interfere with his practice, except so far as to assist, encourage, and support him all we can. If he is a quack; never meddle with his immediate affairs; but give him personally, a severe letting alone. But his doctrines, if they be false, and calculated to do mischief, we should reserve the right to expose whenever occasion may require. Upon the whole, I have an abiding faith in the good sense of the community; the people may be led astray for a while, by the machinations of false pretenders, but this is only for a season; they soon became convinced of the artifice palmed off upon them, and will certainly return to bestow their patronage upon the most deserving.

We are under the strongest and most solemn obligations to the community in which we offer our services as *medical practitioners*, that we be fully qualified and prepared to discharge our duties *as such*. Bear in mind, gentlemen, that our patients are not mere machines, for us to experiment and practice upon. But that they are endowed by nature and by nature's God with the like feelings, sensibilities and reason as ourselves; and that we will be held to a strict account for the manner in which we discharge our duties towards them. In the great day of reckoning, want of knowledge, want of skill, and want of proper attention will not be taken as a valid excuse for homicides committed *secundem artem!* It is our duty to acquire all the knowledge and experience we possibly can, and then to exercise them to the best advantage possible, in every case entrusted to our charge. How dare we, if not properly qualified, take upon ourselves the fearful responsibility of administering medicine to our fellow man? Or attempt to cut with the knife among glands, nerves, tendons and *blood vessels!*—when not properly understanding his anatomical structure? We would necessarily be compelled to grope our way in the dark, and the chances of doing him harm, would be infinitely greater than of doing him any good.

To the community we look for patronage, and the people look to us,—and they have a right to expect of us—proper advice and instructions in relation to the laws of health, as well as medical advice and services. And in these days of 'humbugism,' when almost every day some itinerating doctor or lecturer finds his way among our citizens, for the avowed purpose of enlightening their minds in regard to Fowlerism, Grahamism, and Preisnitzism, and their peculiar rules of hygiene,—it behooves us who profess and of right should be 'guardians of health' in the community, to keep ourselves well informed of the doctrines taught and disseminated among the people by these traveling mountebanks; and, if false, to expose them on all suitable occasions. These *traveling* charlatans are the cause of more disgrace to the 'healing art,' and create more mischief in the community, than all the local quacks, of whatever school they claim to be disciples. For the latter have to undergo the ordeal of the common sense judgment of the community in which they happen to locate, and their ignorant pretentions are sooner or later unmasked; while the former inculcate their peculiar doctrines in the form of popular lectures, and are off before the people have time to measure the *source* from which they received the opinions taught them.

I am one of those who hold the opinion that Grahamism and Preisnitzism have done a great deal of mischief. This thing of feeding the people on chop-bread and black tea; and of wrapping the old and the young, big and little, in wetsheets for every 'ill that flesh is heir to,' has done more harm than is generally acknowledged, and is too often entirely overlooked by physicians. I have not now the time to enter into a full examination of this subject, but suffice it to say—that the effects of these senseless practices may be seen in the sickly-looking, heart-palpitating, half-nourished men, women and children, met with on all occasions, more particularly in neighborhoods where these foolish doctrines most prevail.

But in the enumeration of our duties, Gentlemen, we must not forget those we owe to *ourselves*. We owe it to ourselves, that we build up for us a good reputation, and, in order to do this, we must be good, moral men, we 'must on all occasions feel and act as *gentlemen*.' This is as necessary to our 'advancement in the profession, as knowledge and skill.' A physician may become well versed in the science of medicine, he may be apt and thoroughly skilled in the practice, he may even obtain wealth, and be placed in (erroneously so-called) independent circumstances. But what will all these profit him in the end, if, with all his accomplishments, he is the subject of irregular and immoral habits or vicious practices; the higher, the scientific and professional eminence he has attained, the greater, if he does not immediately reform, will be his fall. He very soon becomes a byword in the community, a disgrace to himself and his friends, and, as a never failing consequence, he will debase the dignity of the profession. There are any number of instances on record; and some, if not all, of us have seen melancholy examples of physicians, who have commenced their career under the most flattering circumstances, and who have for a season gained professional renown and applause; who might have remained ornaments in the profession, and a blessing to the community through life, and died respected and regretted by all; but who have, by their own imprudent and immoral conduct, precipitated themselves from the high eminence which they had attained, into the lowest depths of professional oblivion and moral degradation; and many such have gone down to premature graves—

'Unwept, unhonored, and unsung.'

Let us avoid all immoral and vicious habits as we would the 'bite of a serpent or the sting of an adder.' Let us be resolute and determined, never to be the slaves of any of those evil besetments; which are so directly calculated to impair the faculties of the mind, and which, if they prevent any man from attending to his business in a rational and conscientious manner, have a twofold tendency to incapacitate the physician for his responsible duties, who of all others, should always be in the possession of a clear mind and a steady hand; not knowing what moment he may be called, where life depends upon the manner in which he ministers to his patient!

You will perceive, Gentlemen, how easy it would be, under circumstances such as I have just hinted at, for a physician to sink in an evil hour, a reputation which it had cost him a life-long labor to build up. Let us remember, that 'a good name is rather to be chosen,' than the pleasures and follies of this world!

Before closing, I must remind you that it is now one year since a few of us met for the purpose of organizing a County Medical Society. Some of you, who were present on that occasion, will recollect the remarks I then made, relative to the benefits to be derived, both by physicians and their patrons, from proper medical organizations; and I appeal to you, Gentlemen, if what I said on that occasion has not already been partially verified, so far, at least, as one year can develope the effects of a measure of this kind. We have met four times during the year; have interchanged ideas with one another, which has in a marked degree promoted social feeling among us. We have had essays written, and read before this Society. We have also been favored, to a certain extent, with reports of cases and observations made at the bedside; and I am free to say that the writing and reading of these essays and reports, and the debates they called forth from among us, has been the cause of more searching among authorities, and more original thinking on subjects connected with medicine, in this one year gone by, than would have been the case in a half-a-dozen years, without this *stimulus* to medical emulation.

In conclusion, I must entreat you never to lose the interest I am sure you all now feel in our County, State, and National Societies; attend all the meetings of these bodies you possibly can; and my word for it, you will always return home benefitted. These meetings are not alone useful in promoting the welfare of the profession at large, but also in stimulating to increased individual efforts. Always bear in mind, that associated effort will frequently attain, what individual exertion, however well directed, could never accomplish.

